# Chronic digital infection presenting with gross enlargement of the toes: two case reports and review of the literature

**DOI:** 10.1186/1757-1626-2-102

**Published:** 2009-01-29

**Authors:** Stephen J Cooke, Howard Davies, Nick J Harris

**Affiliations:** 1 Robert Jones & Agnes Hunt Orthopaedic Hospital, Oswestry, Shropshire, SY10 7AG, UK; 2 Leeds General Infirmary, Great George Street, Leeds, LS1 6EX, UK

## Abstract

There are many conditions ranging from the benign to the malignant, which can present with enlargement of one or more digits. An understanding of the differential diagnosis is important such that the potentially serious aetiologies are not missed and patients can therefore be treated appropriately.

We present two patients, a male and a female aged 58 and 49 respectively, who presented to orthopaedic surgeons with gross enlargement of the toes. There were significant delays to presentation in both cases. Histological and microbiological analysis revealed that chronic, untreated infection was the most likely cause in both cases. Both patients were successfully treated by amputation of the offending digits.

Congenital, infective, inflammatory and neoplastic conditions may all cause enlargement of a digit. The cause should be thoroughly sought prior to deciding upon management. Amputation can be successful, enables definitive tissue diagnosis and allows quick return to normal activities. The correct level must be identified pre-operatively.

## Introduction

Enlargement of one or more toes is a rare presentation and therefore detailed knowledge regarding the differential diagnosis is often lacking amongst physicians and surgeons. As described below, patients may present very late and travel through multiple specialties before a definitive diagnosis is found and treatment commenced. It is well taught in medical school how the fingers can be clues to systemic disease. Digital enlargement is no exception and systemic diseases such as inflammatory arthritis, diabetes, gout and hyperlipidaemia may all present in this way. Swelling or enlargement anywhere in the body should raise the possibility of neoplasia (it usually will in the mind of the patient). Both primary and secondary malignancies have been described in the toes. We believe that a working protocol for managing such patients is important to avoid missing a these potentially serious diagnoses.

## Case presentations

### Case 1

**Figure 1:**
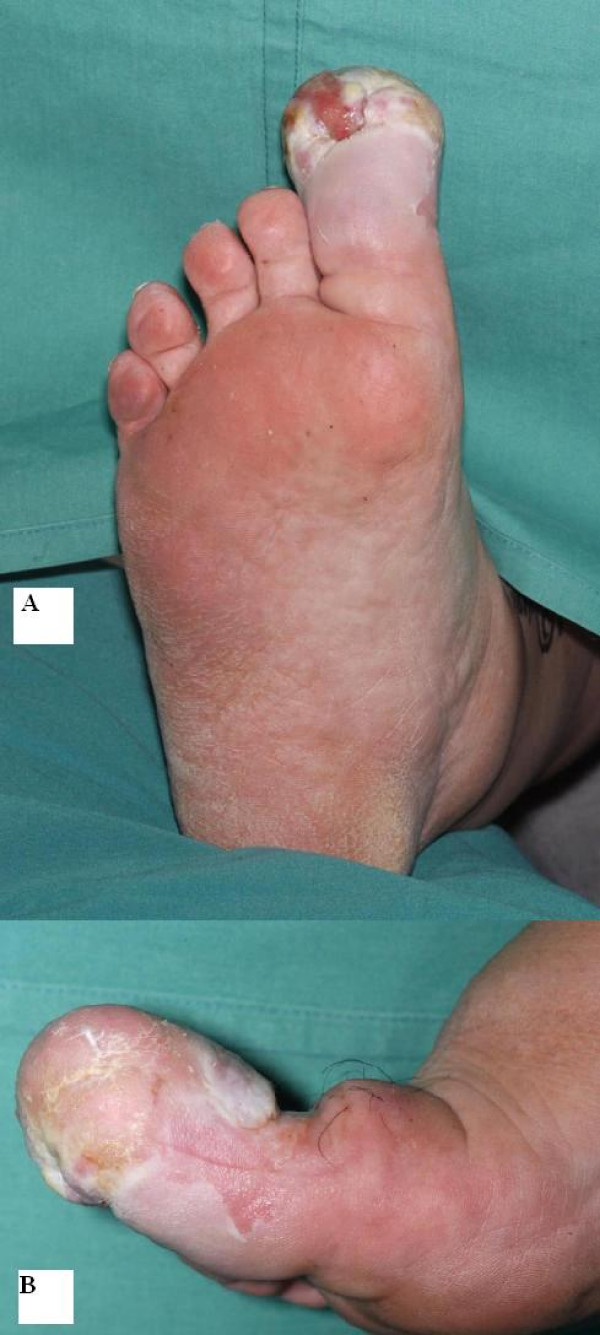
**a AP photograph at the time of surgery (case 1)**. The nail has been completely overgrown by a large mass of tissue and the whole digit is grossly enlargerd. b Lateral photograph of the same toe. Chronic skin changes are visible and the swelling can be seen to extend to the level of the MTPJ.

**Figure 2:**
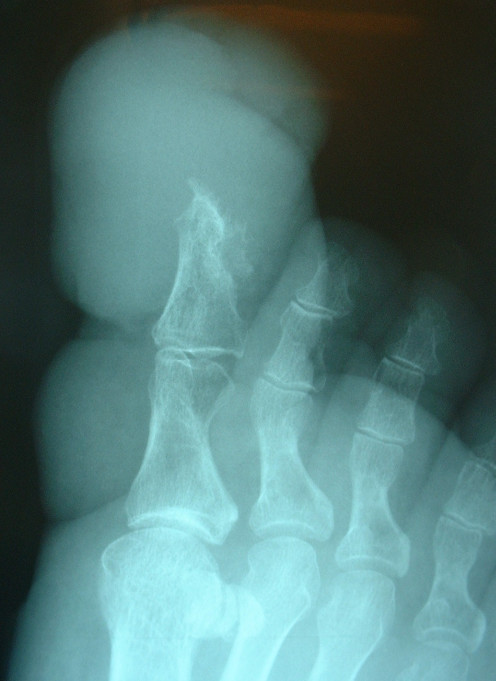
**Plain oblique radiograph of the great toe (case 1)**. The size of the soft tissue swelling can be appreciated compared to the lesser toes and the distal part of the terminal phalanx has been eroded. There is also some calcification within the soft tissues volar to the distal phalanx.

**Figure 3:**
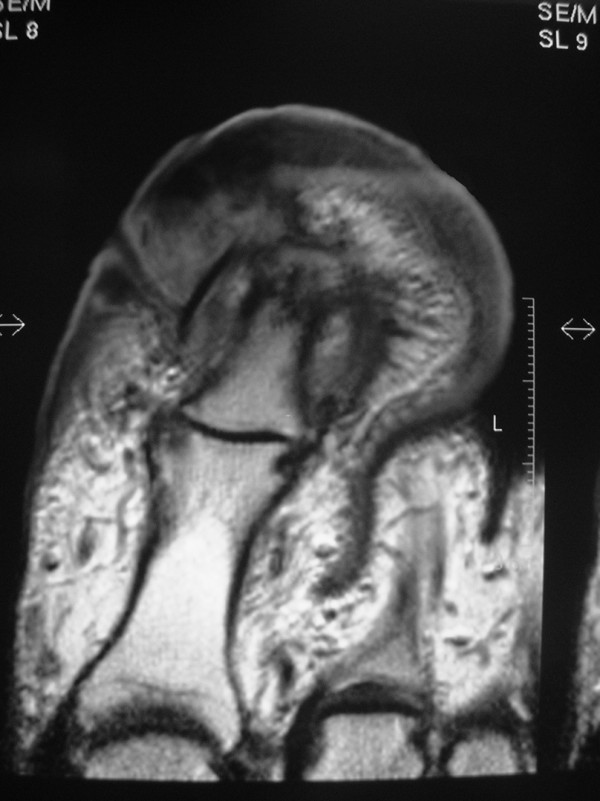
**MRI scan of the great toe (case 1)**. As in the plain film, the end of the distal phalanx has been completely destroyed. There is signal change in the distal part of the proximal phalanx suggesting involvement of the bone more proximally.

A 58 year old, very overweight, type II diabetic lady presented initially to her general practitioner with a thirteen year history of a progressively enlarging right great toe following a small laceration. The reason for the delayed presentation was attributed to the patient's fear of hospitals and it was her partner who encouraged her to eventually seek medical advice. The toe was measured at approximately 5 cm wide by 8 cm long. The nail was almost completely buried under a mass of inflamed tissue and the toe was particularly foul smelling (figure 1). There was no abnormality of the foot proximal to the first MTPJ, which had a full and pain free range of movement, sensation was intact and peripheral pulses were good. She was initially referred to the general surgeons who felt there was no significant peripheral vascular disease. Plain x-rays done at that time showed some bony erosion of the distal phalanx of the great toe and obvious soft tissue swelling (figure 2). Some basic blood tests, including white cell count and c-reactive protein were normal. She was then referred to an orthopaedic surgeon as chronic infection ± osteomyelitis. An MRI scan was requested to determine the extent of the bony involvement and soft tissue disease (figure 3). This identified gross peri-ungual soft tissue swelling with little, if any, involvement of the distal phalanx and no abnormality proximal to the IPJ. There was marked enhancement post gadolinium and the radiologist concluded that the swelling was due to chronic infection with no evidence of osteomyelitis but malignancy could not be excluded. She was offered and accepted amputation, which was done through the MTPJ. The wound healed well with no evidence of infection or recurrence.

### Case 2

A 49 year old gentleman presented with a five year history of bilateral great toe swelling. He had previously been troubled with, but had never been treated for, in growing toenails on both feet. Other than controlled hypertension, he was fit and well and able to continue his quite physical profession. Both his toes had intermittently discharged purulent fluid and he felt they were growing in size. On examination he had enormous swelling of both great toes which discharged seropurulent fluid on pressure. Microbiological cultures of this fluid never grew any organisms. Again he had good sensation, pulses and range of movement and again an urgent MRI scan was done. As in case 1, there was marked soft tissue swelling beneath, around and extending over the nail with enhancement after gadolinium, no involvement of the bone was seen and no abnormality extended proximal to the MTPJs. Both of his great toes were amputated through the MTPJ successfully with good healing of the wounds.

Pathologically all 3 specimens contained an abundance of granulation tissue and fibrous scar tissue with gross destruction of the normal architecture. There were no obvious areas of malignancy and no bacteria were cultured.

## Discussion

In both of the above cases it seemed obvious from the outset that there was an infective process as the cause of the extreme swelling. However the cause of and reason for the persistence of infection was less clear. Underlying systemic disease, osteomyelitis or malignancy could all contribute. Indeed, the lady in case 1 was a type II diabetic and chronic infection, particularly of the extremities is well known in this group of patients [1]. Clinical examination, microbiology swabs and MRI scans were all inconclusive and in particular, malignancy could not be categorically excluded. On review of the literature, the number of potential diagnoses for one or more enlarged digits are legion. Conditions can be found within every category of the 'surgical sieve' including infective, inflammatory, neoplastic, traumatic, congenital and metabolic.

In case 1 there is a history of trauma to the toe, although she admits her memory after thirteen years is somewhat hazy. Any trauma to the skin, soft tissues or bone may be complicated by infection. Given that she is also diabetic, this could explain the chronicity of the infection. Osteomyelitis can commonly become chronic if not treated and the history in case 2 of an intermittently discharging wound is typical of this. Also, foreign body granulomas or myositis ossificans following bony injury can cause significant swelling and inflammation [2].

Of the infective causes, chronic paronychia is the commonest [3]. However, fungal and even parasitic infestations have been documented [4]. It must be noted, however, that infection may be associated with other conditions such as diabetes and malignancy. Marjolin's ulcer, where squamous cell carcinoma arises in an area of chronic ulceration is well documented. Underlying infection of the bone or joint must always be suspected.

Inflammatory causes such as tophaceous gout and pseudogout can closely mimic infection [5]. Rheumatoid disease and other inflammatory arthropathies can, rarely, affect distal extremities, as can proliferative fasciitis [2]. Inflammatory markers should be documented and are a useful guide to disease progression.

There are many neoplastic conditions that can affect the toes. Both benign and malignant, primary and secondary tumours have been found at this site. Squamous cell carcinoma as mentioned above is the commonest but others must be taken into account. Due to their rarity, it is difficult from the literature to assess incidence and prevalence. However, both myxohyaline tumours [6] and pleomorphic fibromas [7] have long natural histories and have been mistaken for chronic infection. It is not clear whether either of these tumours have metastatic potential, but both can be locally recurrent if not completely excised. Giant cell tumour of the tendon sheath, the commonest benign neoplasm of synovium, can occur here and this has led some authors to suggest fine needle aspiration cytology may be a useful diagnostic tool prior to planning any definitive treatment [8]. Other primary tumours include lipomas and liposarcomas [12] and indeed, any other sarcomas such as rhabdomyosarcoma, osteosarcoma and chondrosarcoma. The incidence of these occurring in a toe is extremely low. Metastases to the toes are also rare, however, one case report of note documented a metastasis to the great toe from an endometrial carcinoma presenting with similar features to those in this report [9]. Secondary tumours in the foot, as elsewhere in the skeleton, tend to arise from colorectal, lung, kidney and prostate tumours. The tarsal bones, especially the calcaneum, are most commonly involved, but more distal spread to the toes has been observed [9].

Systemic disease must always be sought when assessing the patient with chronic or recurrent infections. Diabetes is by far the commonest finding in these cases. As mentioned above, rheumatoid disease can affect the toes. Other systemic conditions that have been noted to cause enlargement of one or more digits are tendon xanthoma due to hyperlipidaemia and tuberous sclerosis [10]. Certainly screening tests for diabetes and rheumatoid should be performed.

Finally, in a young patient, congenital causes must be considered. Macrodactyly can occasionally be isolated or occur in conjunction with other syndromes such as neurofibromatosis, Milroy's disease, Klippel-Trenaunay-Weber syndrome and Proteus syndrome [11].

## Conclusion

In the light of the above differential diagnoses and in particular, the risks of malignancy, it is important to treat such cases thoroughly and with a reasonable degree of urgency. Both of these patients were scanned and operated on within one month of presentation. The use of FNA cytology, provided the general principles are followed correctly, may yield a diagnosis, but a negative result for malignancy cannot definitely exclude this. We believe that in these two cases of quite bizarre toe enlargement there was no option for definitive diagnosis or cure other than amputation. MRI was used to determine the extent of disease and therefore the level of amputation and the specimens were sent for pathological and microbiological analysis. Both patients have recovered well and are now back to their normal levels of activity.

## Competing interests

The authors declare that they have no competing interests.

## Consent

Written informed consent was obtained from both patients for publication of this case report and accompanying images. A copy of the written consent is available for review.
